# Supplementary material for: Comparative Analysis of Inhibitory and Activating Immune Checkpoints PD-1, PD-L1, CD28, and CD86 in Non-Melanoma Skin Cancer
Source: Cells. 2024 Sep 18;13(18):1569. doi: 10.3390/cells13181569 (PMC11430031; doi:10.3390/cells13181569)
Supplement: Supplementary file 1 [file cells-13-01569-s001.zip › cells-3069343-supplementary.pdf]

Supplementary Figure S1. Expression of PD-1, CD28, PD-L1 and CD86 in BCC and cSCC by stroma labeling indices

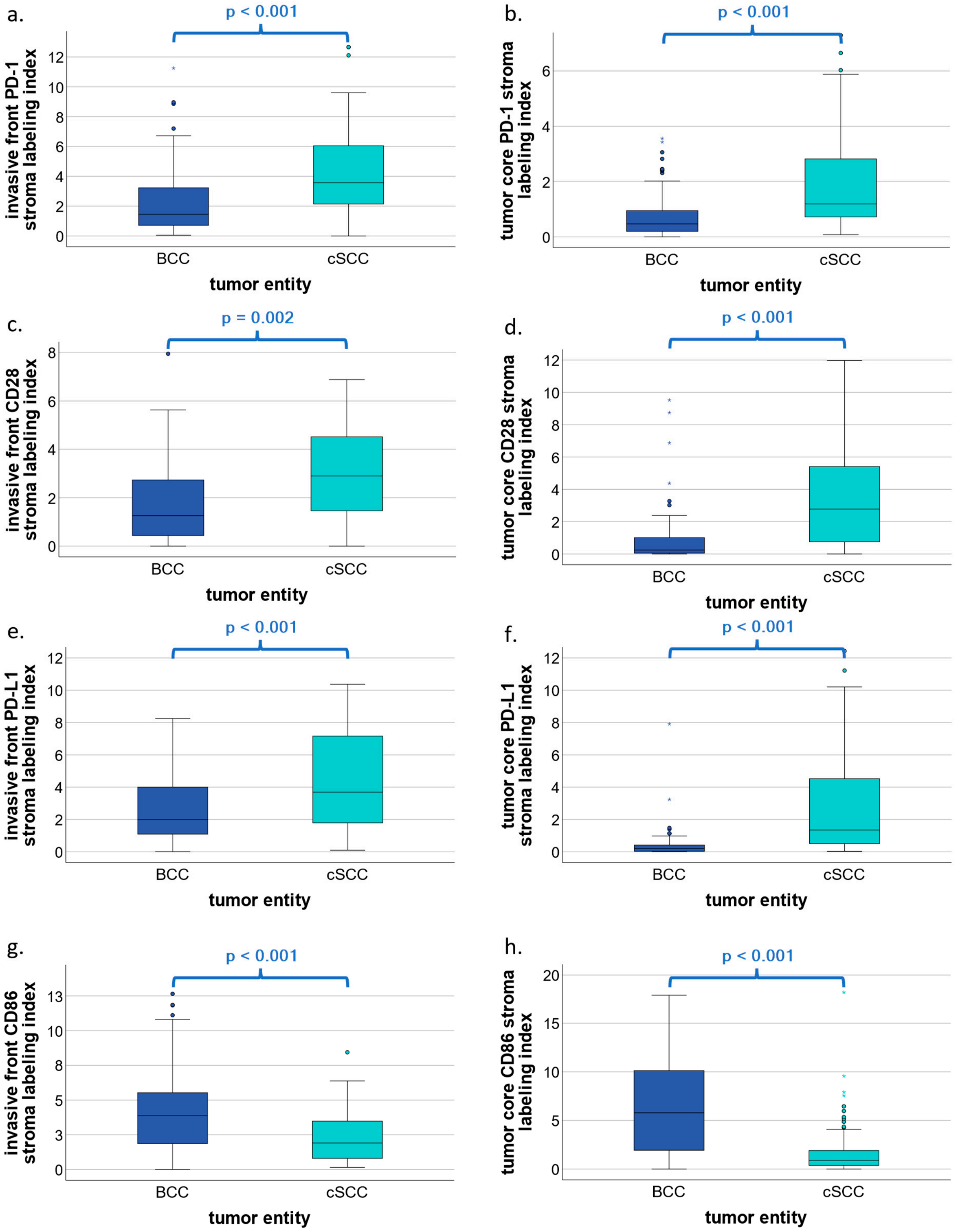

Box plots show PD-1, CD28, PD-L1 and CD86 expression in basal cell carcinoma (BCC) and cutaneous squamous cell carcinoma (cSCC) (a-h): Box plots show PD-1, CD28, PD-L1, and CD86 expression in the invasive front and the tumor core. Stroma labeling indices include only cell counts from stromal

cells within the invasive front or tumor core. P values were calculated using the Mann-Whitney U test.

Supplementary Table S1. Comparison of PD-1/PD-L1, CD28/CD86, PD-1/CD28, PD-L1/CD86 ratio means, SD and p-values by total cell labeling indices

		invasive front		tumor core	
ratios		BCC	cSCC	BCC	cSCC
PD-1/PD-L1	n	57	47	66	84
	mean	1.54	0.58	6.68	2.37
	SD	2.33	0.65	10.39	4.43
	p-value	**p < 0.001**		**p < 0.001**	
CD28/CD86	n	58	41	60	50
	mean	0.87	3.40	0.38	15.66
	SD	1.98	4.95	0.99	46.01
	p-value	**p < 0.001**		**p < 0.001**	
PD-1/CD28	n	57	44	52	45
	mean	4.30	3.96	2.37	1.41
	SD	7.28	8.59	4.25	2.44
	p-value	p = 0.962		**p = 0.028**	
PD-L1/CD86	n	52	42	64	89
	mean	0.83	18.63	0.23	124.36
	SD	0.89	27.40	0.93	734.10
	p-value	**p < 0.001**		**p < 0.001**	

Values represent number of cases (n), median, standard deviation (SD), and p-value (Mann-Whitney U test) of marker ratios in basal cell carcinoma (BCC) and cutaneous squamous cell carcinoma (cSCC). Total cell labeling indices include cell counts of both tumor epithelial and stromal cells within the invasive front or tumor core. Significant (p≤0.05) and highly significant (p≤0.001) values are shown in bold.

Supplementary Table S2. Comparison of expression patterns in the invasive front and tumor core of PD-1, CD28, PD-L1 and CD86 in BCC and cSCC by total cell labeling indices

		BCC		CSCC	
marker		invasive front	tumor core	invasive front	tumor core
PD-1	n	69	69	50	85
	mean	1.91	0.50	4.00	1.42
	SD	2.04	0.65	4.80	1.69
	p-value	**p < 0.001**		**p < 0.001**	
CD28	n	64	65	47	54
	mean	2.21	0.88	2.73	3.50
	SD	3.50	2.22	3.08	4.98
	p-value	**p < 0.001**		p = 0.752	
PD-L1	n	64	72	48	103
	mean	1.93	0.26	11.90	5.20
	SD	1.63	0.39	10.24	10.62
	p-value	**p < 0.001**		**p < 0.001**	
CD86	n	66	71	45	95
	mean	3.28	3.18	1.66	0.98
	SD	3.06	2.49	1.47	1.15
	p-value	p = 0.899		**p = 0.001**	

Comparison of marker expression in the invasive front versus the tumor core in basal cell carcinoma (BCC) and cutaneous squamous cell carcinoma (cSCC). Total cell labeling indices include cell counts of both tumor epithelial and stromal cells within the invasive front or the tumor core. Number of cases (n) and standard deviation (SD). P-values were generated using the Mann-Whitney U test.
